# Host diversity of *Aedes albopictus* in relation to invasion history: a meta-analysis of blood-feeding studies

**DOI:** 10.1186/s13071-024-06490-4

**Published:** 2024-10-03

**Authors:** László Zsolt Garamszegi

**Affiliations:** 1 Institute of Ecology and Botany, HUN-REN Centre for Ecological Research, Alkotmány u. 2–4, 2163 Vácrátót, Hungary; 2 National Laboratory for Health Security, HUN-REN Centre for Ecological Research, Budapest, Hungary

**Keywords:** Ecological niche, Emerging infectious diseases, Host range, Human-biting rate, Literature review, Vector competence

## Abstract

**Background:**

The invasive mosquito *Aedes albopictus* is a major concern for human and animal health given its high potential to spread over large geographical distances, adapt to various habitats and food sources, and act as a vector for pathogens. It is crucial to understand how this species establishes ecological relationships at different locations, as it determines its role in transmission of diseases.

**Methods:**

Based on published blood meal surveys, a meta-analysis was performed to investigate how host diversity changes along the process of invasion at a large scale. For 48 independent localities, the Shannon diversity index was calculated and was then assessed against several moderator variables describing invasion status, habitat type, methodology, survey year and the year of introduction for invasive populations.

**Results:**

Diet diversity was higher in the invasive than in the native populations when the strong habitat effects were held constant. Furthermore, the year of introduction also had a significant role, as invasive populations that had been established earlier had wider diet diversity than more recent populations.

**Conclusions:**

Invasive *Ae. albopictus* has considerable ecological flexibility. The species’ ability to adapt to various food sources goes hand in hand with its successful worldwide dispersion, which has strong implications for its role in pathogen transmission.

**Graphical Abstract:**

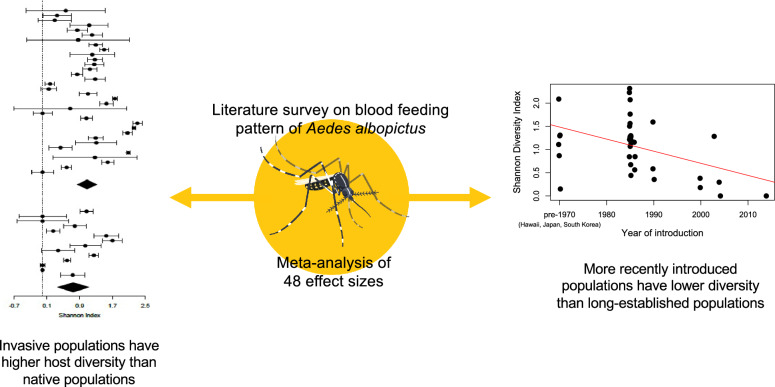

**Supplementary Information:**

The online version contains supplementary material available at 10.1186/s13071-024-06490-4.

## Background

The spread of the invasive mosquito *Aedes albopictus* poses a global challenge, as it is a potential vector of several emerging and endemic pathogens (including viruses, protozoans, and filarial nematodes) with zoonotic and veterinary importance [1, 2]. The role in disease transmission depends on the taxonomic range of hosts with which the mosquito comes into contact through blood meals. [3]. If the vector has a diverse host range, its diet is more likely to include reservoir and amplification hosts, thereby increasing its capacity to transmit parasites among distantly related host species.

Several studies have characterized the blood-feeding patterns of *Ae. albopictus* and identified its vertebrate hosts in both the native and invasive range of the species [reviews in: 4, 5, 6, 7]. These blood meal analyses indicated that this mosquito feeds primarily on humans, but the diet also includes other vertebrate species. Due to its opportunistic feeding behavior, it can be considered a typical bridge vector linking zoonotic arboviruses and humans [e.g. 4, 5]. These studies also demonstrate remarkable variation in the taxonomic composition of the diet across localities, which can be explained by the difference in the availability of different food sources at the sampling sites [6, 7]. Therefore, patterns emerging from blood meal analyses are more likely to reflect the aspects of the mosquito–host associations that are influenced by the environment and diet plasticity than genetically inherited host preference [8]. Accordingly, the large percentage of human-derived blood meals is the consequence of studies focusing on urbanized areas that offer optimal breeding opportunities but also incur inherently high contact rates with humans.

The Asian tiger mosquito, *Ae. albopictus*, is native to Southeast Asia, where it originally inhabited forests and forest edges, breeding in tree holes and other small natural reservoirs [9, 10]. This species has adapted well to urban and suburban environments due to the larvae's ability to develop in artificial breeding sites such as water containers, tires, saucers, and other similar habitats. This aspect, together with the intensification of human trade and tourism, has facilitated its spread around the world [11]. The differences in the breeding habits between native and invasive distribution ranges may mediate dissimilarities in the ecological niches that populations occupy in these areas [12, 13]. This may also have consequences for the diet's taxonomic spectrum and the role played in pathogen transmission. Previous literature reviews [6, 7] suggested that in its native range, the proportion of blood meals derived from human hosts is lower than in the invaded distribution area, probably because sampling in the native range covered more rural sites.

Here, by conducting a meta-analysis on the available evidence on host-feeding patterns, I investigate quantitatively the effect of global distribution on the ecological relationships of this key invasive species. Specifically, I explore whether the diversity of taxa in the diet is different between native and invasive ranges. I predict that range expansion at the global level has given rise to a contact rate with a broad spectrum of hosts, which would lead to higher taxonomic diversity of the diet in the invasive zone than in the native zone. Furthermore, I predict that more recent introductions would result in a narrower host range than earlier introductions, because the shorter time frames since the establishment allow fewer interactions with diverse habitats and hosts.

## Methods

### Literature survey and calculation of Shannon diversity

A literature search using the Web of Science Core Collection database (until 09/08/2024) was performed based on a combination of keywords (i.e., “*Aedes albopictus*,” “blood hosts,” “blood meal,” “blood feeding,” “blood analysis,” “host identification,” “host preference,” “host choice,” “feeding pattern”). These searches identified 382 records, and this set was further filtered by following the Preferred Reporting Items for Systematic Reviews and Meta-Analyses (PRISMA) guidelines [14]. The selection process resulted in 35 independent data sources (Supplementary information, Figure S1), which were cross-validated with references from previous reviews [6–8, 15]. Two additional relevant studies were suggested by a colleague/reviewer. Eight of the source studies presented data for distinct localities; thus, outputs from these were considered separately, leaving 48 entries to be used in the meta-analysis (Supplementary information, Dataset S1).

The results of the blood meal analyses typically listed the number of individual mosquitoes fed on different taxa/species. For each output, I counted incidences for the following typically screened taxonomic categories: human, dog, cat, fox, rabbit, swine, goat, sheep, rat, deer, raccoon, horse, cow, opossum, monkey, mongoose, murids, vole, squirrel, bat, other mammal, bird, reptile, amphibian, and fish. When no count data were reported for any of these categories, they were considered as 0 counts, if it could be verified that the given taxon was truly tested but no incidence was found (e.g., all polymerase chain reaction [PCR] surveys with sequencing, and if it was specifically stated in studies using the precipitin test and enzyme-linked immunosorbent assay [ELISA]); otherwise it was considered as missing information (NA). From the tabulated data, I calculated the Shannon diversity index and its variance for each output to reflect species diversity in the blood meal and its precision, respectively [see 16]. This metric was chosen because this is the simplest and most commonly used estimate of diversity accounting for abundance that could be robustly calculated across studies varying in data quality and quantity.

### Moderator variables

The source studies relied on different laboratory techniques to characterize blood content, and thus entries were categorized based on the underlying methodology (i.e., precipitin test, ELISA, PCR). I also extracted the median year of the field surveillance from which the analyzed samples originated. To calculate the distance matrix of sampling localities, the geographical coordinates of the field sites were determined as precisely as the information in the source paper permitted. Habitat was coded along the following criteria: nature—extended, non-inhabited areas with natural, or semi-natural vegetation and with accidental human presence; rural sites—areas with sparse human habitation and high vegetation coverage including farms and small villages; peri-urban sites—areas with intermediate population density and with some remnant vegetation, residential suburban areas with gardens, city parks, zoos, city-nature interface; and urban sites—thickly populated areas without vegetation, city centers, highways. This coding was done based on the detailed site descriptions provided in the source paper (if this was not available, the categorization in the source paper was followed). Each entry was assigned according to the species' status at the global scale (native or invasive ranges). For each country in the invasive range, the year of introduction was determined based on the information in the source paper, or from other literature sources as needed [e.g., 17, 18]. Only Southeast Asian regions were considered as part of the native range; Japanese and South Korean populations were treated as part of the invasive range (with introductions in the 1700s and in 1940, respectively [19, 20]).

### Statistical analyses

A meta-analytic framework for linear mixed-effects models [21] was applied to test whether the taxonomic diversity of the diet depends on the distribution status of the species by using the *metafor* R package [22]. The model included the focal effect size, Shannon diversity index as the response variable, and the following moderator variables as predictors: distribution status, habitat, year of sampling, and underlying methodology. Study identity was included as a random effect to control for the fact that in some cases multiple effect sizes were calculated from the same studies. The variance in the calculated Shannon diversity index values was used as a sampling error variance to define the random part of the model. To account for the spatial non-independence of data (some effect sizes originate from neighboring countries, while others are separated by larger distances), the random effects were forced to follow a Gaussian spatial correlation structure based on the distance matrix of study sites. The significance of categorical moderators was determined by the likelihood ratio test (LRT), in which the full model was contrasted with the model lacking the moderator of interest.

I also investigated whether the year of introduction could affect the detected taxonomic diversity of the diet by using data for the invasive populations. In the corresponding meta-analytic model, the Shannon diversity index was the response variable, and habitat, year of introduction, year of sampling, and methodology were the predictors. The random part of the model was defined as above.

## Results

**Fig. 1:**
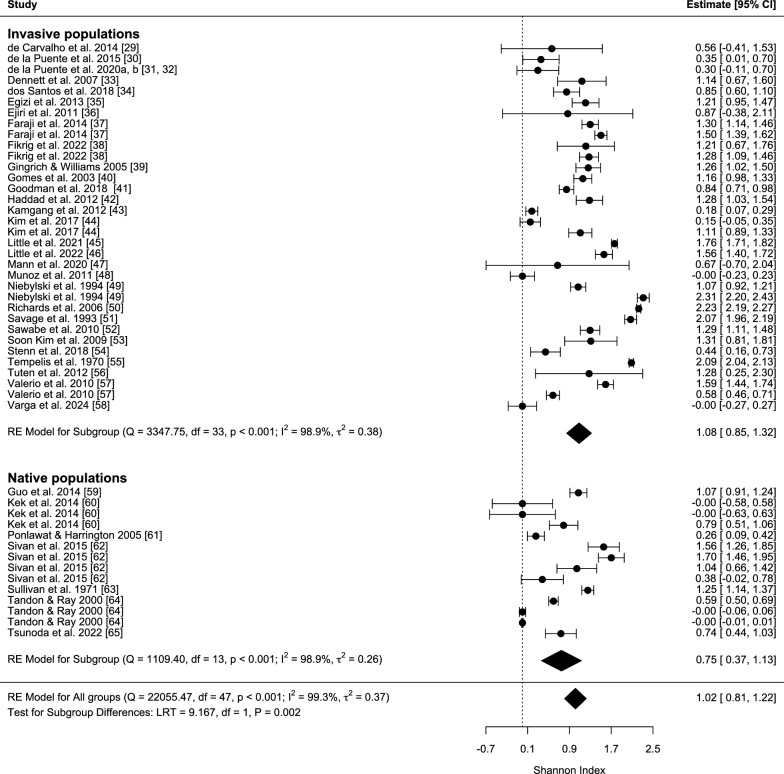
Meta-analytic summary of studies testing for the diversity of diet composition of the *Aedes albopictus* mosquito. From the outputs of the blood meal analyses, the Shannon diversity index was calculated for each source study, and these we tabulated separately for invasive and native populations. For each population, the calculated Shannon diversity index is shown (black dots) together with its estimated 95% confidence interval (error bars). Black diamonds indicate the overall mean effect sizes that are calculated over different groups of studies (invasive populations, endemic populations, all studies)

**Fig. 2:**
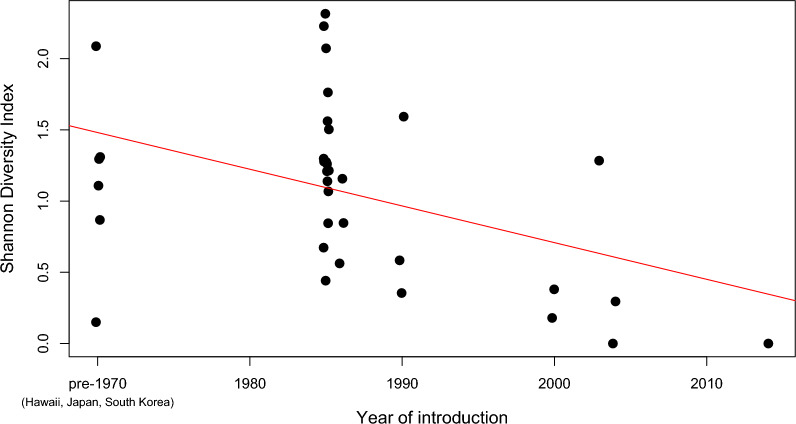
Relationship between the year of introduction and the host diversity as could be assessed from blood meal patterns for the invasive populations of *Aedes albopictus*. Points represent estimates from particular studies and are jittered along the *x*-axis for better visualization. To avoid the influence of few data points with very early introductions, the year of introduction variable was truncated at 1970. The line is the meta-regression line from a meta-analysis that also controlled for the moderator variables and the considered random effects (slope estimate ± standard error (SE) for year of introduction = −0.038 ± 0.012, *P* = 0.002; after excluding the Hawaiian, Japanese, and Korean populations with introductions before World War II: slope estimate ± SE for year of introduction = −0.041 ± 0.014, *P* = 0.003)

The mean diversity of the diet was smaller in the populations within the native range of the species than in the invasive populations (Fig. 1). Importantly, this difference persisted even after statistically accounting for the moderator variables considered in the model, among which the effect of habitat was highly significant (Table 1). When considering invasive populations only, there was a negative relationship between the year of introduction and Shannon diversity index, indicating that more recently introduced populations had a narrower host range (Fig. 2).

**Table 1:** Results of linear mixed-effects meta-analysis model with spatial autocorrelation testing for the effect of distribution status on the taxonomic diversity of blood meals in *Aedes albopictus*

Variable	Estimate	SE	*z*	*P*	95% CI
Intercept	42.349	23.65	1.791	0.073	−4.004/88.703
Status [invasive]	0.825	0.276	2.996	0.003	0.285/1.365
Method [PCR]	0.012	0.277	0.044	0.965	−0.530/0.555
Method [precipitin test]	0.183	0.392	0.467	0.641	−0.586/0.952
Habitat [peri-urban]	0.012	0.102	0.114	0.909	−0.189/0.212
Habitat [rural]	0.845	0.111	7.616	< 0.001	0.627/1.062
Habitat [urban]	−0.167	0.103	−1.625	0.104	−0.368/0.034
Sample year	−0.021	0.012	−1.775	0.076	−0.044/0.002

*SE* standard error

Significance of categorical moderators: status, LRT = 9.167, *P* = 0.003; method, LRT = 0.262, *P* = 0.877; habitat, LRT = 305.05, *P* < 0.001

*Q* statistics: *Q*_M_ = 315.5, *df* = 7, *P* < 0.001, *Q*_E_ = 3299.0, *df* = 40, *P* < 0.001

## Discussion

The results were in accordance with the prediction that higher taxonomic diversity in the diet should be observed in the invasive populations of *Ae. albopictus* than in the native distribution range. This prediction was based on the ecological premise that dispersing populations can undergo niche shifts, enhancing the rapid spread over geographical ranges and the successful colonization of different habitats [12, 13]. This link between range expansion and ecological flexibility has been shown in various animal taxa [21]. The fact that native populations of *Ae. albopictus* originally breed in tree holes while invasive populations rely more on artificial containers in the urbanized environment [9, 10] already suggests that this species can occupy different ecological niches in different parts of its distribution range. Furthermore, it is also known that in the invaded temperate zones, the populations have developed effective strategies to successfully survive the winters that are much colder than in their native zone [23–25]. For example, they evolved an ability to produce diapause eggs, thrive in urban environments, leaving behind their dependence on the natural reservoir forest cycles, and maintain breeding activity all year-round [26, 27]. The current meta-analytical findings highlight that the ecological plasticity associated with rapid geographical dispersion also includes a flexible adaptation to a broad range of food sources. Such niche shift may result either from the species exploiting a greater part of its fundamental niche during the invasion or from the rapid evolution of traits favoring the species to acclimatize to novel environmental conditions in the introduced range [28].

The results also support the prediction that more recent introductions should be associated with a narrower host range than introductions in the more distant past. This finding indicates that wider taxonomic diversity of the diet is not the cause but the consequence of expanding invasion ranges. If an established population has sufficient time to exploit the available food resources, it will be able to incorporate these in its diet. Therefore, the detected niche shift can be a result of the species exploiting a greater part of its fundamental niche as time progresses. This may mean that invasive populations that have been established for a longer time have a higher potential to transmit pathogens among distantly related hosts than more recently introduced populations.

The meta-analytic model also revealed a strong role for habitat type mediating differences in host diversity (e.g., mean Shannon index for urban habitats was considerably lower than for rural habitats). Habitat is a well-known factor that mediates detected feeding patterns [6, 7], and supporting evidence for this relationship is often used as an argument for the flexible adjustment of the diet according to the available food sources [8]. A high proportion of human blood in the urban samples leaves lower percentages for the other animal taxa being represented in the diet, which altogether results in a small diversity index. This is in line with the results of this study with regard to the habitat effects. However, it is important to emphasize that the main differences between introduced and native populations are independent of these habitat effects (Table 1). Therefore, the higher host diversity observed in the native range is not the consequence of mosquitoes originating from a greater diversity of habitats that expose them to a wider range of food resources, but it should reflect a more direct link between colonization success and diet flexibility.

## Conclusions

The results have strong implications for how *Ae. albopictus* mediates host–parasite dynamics in natural systems. Wider host diversity in the invasive range indicates that the chances for the species to act as a bridge vector are higher than in the native distribution range, and this risk further increases if the species has more time to adapt to the ecological conditions experienced in a given invaded region. For example, a larger Shannon index implies a larger proportion of bird-originated blood in the diet (*r* = 0.380, *P* = 0.013). Accordingly, the chances of transmitting parasites between humans and other vertebrates are higher in zones where species diversity in the diet is also higher. Therefore, the obtained results can align with the ecological foundations that make this species a widespread disease vector worldwide. Given the correlative nature of the study, further experimental studies are needed that can identify the mechanistic link between invasion status, food preference, and the role played in pathogen transmission.

## Supplementary Information

Additional file 1: Dataset S1. Source data used in the meta-analysis together with the corresponding references.

Additional file 2: Figure S1. PRISMA diagram showing the selection process of source studies from the results of a literature search based on keywords.

## Data Availability

All data generated or analyzed during this study are included in this published article [and its supplementary information files.
