# Supplementary material for: Host diversity of Aedes albopictus in relation to invasion history: a meta-analysis of blood-feeding studies
Source: Parasit Vectors. 2024 Oct 3;17:411. doi: 10.1186/s13071-024-06490-4 (PMC11448256; doi:10.1186/s13071-024-06490-4)
Supplement: Supplementary file 2 — Additional file 2: Figure S1. PRISMA diagram showing the selection process of source studies from the results of a literature search based on keywords. [file 13071_2024_6490_MOESM2_ESM.docx]

**Supplementary Information**

**Figure S1.** PRISMA (Preferred Reporting Items for Systematic Reviews and Meta-Analyses) flow diagram reporting the number of records identified and excluded during the literature search process.

**Abstract and Title screening**

**Full text screening**

**Relevant studies for blood meal analysis in *Ae. albopictus***

37 articles

Number of countries: 14 (5 endemic, 9 invasive)

Total effect sizes: 48

**Studies excluded**

18 articles

**Unable to source full text**

0 articles

**Language restrictions**

0 articles

**Not relevant data, not included taxonomic outcomes, laboratory studies**

**Studies excluded**

255 articles

**Duplicates excluded**

52 articles

**Reviews excluded**

22 articles
